# P04.88. Education plus exercise vs. education, exercise and chiropractic care for veterans with chronic low back pain: a pilot randomized trial

**DOI:** 10.1186/1472-6882-12-S1-P358

**Published:** 2012-06-12

**Authors:** H Fink, C Schulz, A Bangerter, S Frizelle, A Baines-Simon, S Noorbaloochi, G Bronfort

**Affiliations:** 1 Veterans Affairs Medical Center, Minneapolis, USA; 2 Northwestern Health Sciences University, Bloomington, USA

## Purpose

Little is known regarding the effectiveness and safety of chiropractic therapy for chronic low back pain (LBP) in veterans. This collaborative project integrated conventional and complementary alternative medicine (CAM) researchers to conduct a pilot study investigating feasibility of a full-scale trial of chiropractic therapy in this population.

## Methods

Veterans were recruited within the Minneapolis VA Medical Center, through flyers given to LBP patients, recruitment posters, and direct mailings to patients diagnosed with LBP. All participants were screened at the VA Medical Center and interventions were delivered at Northwestern Health Sciences University. After screening, eligible participants were randomized to education plus exercise (EE) vs. education, exercise and chiropractic treatment (EEC). EE participants also were scheduled for assessment visits to balance contact time. Outcomes were ascertained by mailed questionnaires.

## Results

Recruitment Feasibility: The pool of local enrolled veterans eligible for screening from these sources was estimated at >40,000. 1075 veterans were offered initial phone screening, 71 completed in-clinic screening, and 30 were randomized.

Participant Protocol Adherence: 93% of participants completed ≥ 3 of 4 scheduled education plus exercise sessions. 90% of EEC participants completed ≥12 chiropractic visits. 60% of EE participants completed ≥ 6 of 8 scheduled assessment visits. Mailed questionnaire completion was 70%.

## Conclusion

The integration of conventional and CAM researchers successfully demonstrated feasibility of a full-scale trial. Recruitment yield was generally high, with the patient pool potentially eligible for screening far exceeding that needed to enroll an adequately powered trial. Adherence with intervention visits was high. Protocol refinement will address lower than desired adherence to assessment visits and mailed questionnaires.

